# Error and bias in size estimates of whale sharks: implications for understanding demography

**DOI:** 10.1098/rsos.150668

**Published:** 2016-03-23

**Authors:** Ana M. M. Sequeira, Michele Thums, Kim Brooks, Mark G. Meekan

**Affiliations:** 1 IOMRC and The UWA Oceans Institute, School of Animal Biology and Centre for Marine Futures, University of Western Australia (M470), 35 Stirling Highway, Crawley, Western Australia 6009, Australia; 2 School of Civil, Environmental and Mining Engineering and UWA Oceans Institute, University of Western Australia (M470), 35 Stirling Highway, Crawley, Western Australia 6009, Australia; 3 Australian Institute of Marine Science, c/o UWA Oceans Institute (MO96), 35 Stirling Highway, Crawley, Western Australia 6009, Australia

**Keywords:** stereo-video measurements, migratory species, *Rhincodon typus*, maturity, coastal aggregation, conservation strategies

## Abstract

Body size and age at maturity are indicative of the vulnerability of a species to extinction. However, they are both difficult to estimate for large animals that cannot be restrained for measurement. For very large species such as whale sharks, body size is commonly estimated visually, potentially resulting in the addition of errors and bias. Here, we investigate the errors and bias associated with total lengths of whale sharks estimated visually by comparing them with measurements collected using a stereo-video camera system at Ningaloo Reef, Western Australia. Using linear mixed-effects models, we found that visual lengths were biased towards underestimation with increasing size of the shark. When using the stereo-video camera, the number of larger individuals that were possibly mature (or close to maturity) that were detected increased by approximately 10%. Mean lengths calculated by each method were, however, comparable (5.002 ± 1.194 and 6.128 ± 1.609 m, s.d.), confirming that the population at Ningaloo is mostly composed of immature sharks based on published lengths at maturity. We then collated data sets of total lengths sampled from aggregations of whale sharks worldwide between 1995 and 2013. Except for locations in the East Pacific where large females have been reported, these aggregations also largely consisted of juveniles (mean lengths less than 7 m). Sightings of the largest individuals were limited and occurred mostly prior to 2006. This result highlights the urgent need to locate and quantify the numbers of mature male and female whale sharks in order to ascertain the conservation status and ensure persistence of the species.

## 1. Background

Anthropogenic impacts such as overexploitation by fisheries, habitat loss and pollution are promoting the rapid loss of marine biodiversity [1,2] and driving changes to the structure of marine food webs [3,4]. Because many large marine vertebrates have *K*-selected life-history traits (i.e. slow growth, late age of maturation and long life expectancy), these species are particularly susceptible to human impacts [1,5], and there is increasing concern over the status of their populations [6]. However, tracking the demographic trends of marine megafauna can be problematic as many (e.g. whales and sharks) spend the majority of their lives beyond coastal shelves in the open ocean [7,8] where sampling is difficult and expensive [9]. Hence, available datasets are often spatially and temporally incomplete, making estimation of abundance trends controversial [10]. This lack of data complicates the assessment of both the impacts of human activities and strategies that aim to appropriately manage and conserve populations of megafauna [11,12].

These problems characterize studies of the ecology of the world's largest fish, the whale shark (*Rhincodon typus*, Smith 1828). Whale sharks are a migratory species, with populations possibly connected at a global scale [13], but with some genetic evidence for dissimilarities between the populations from the Atlantic and Indo-Pacific Oceans [14–16]. Following previous declines in catches of targeted fisheries [17], whale sharks were classed as vulnerable in 2000 by the International Union for the Conservation of Nature (IUCN) Red List (www.iucnredlist.org), and the same status was kept in more recent assessments. Although many countries have now banned directed fisheries and afforded protected status to the species (IUCN Red List; [18]), there are numerous lines of evidence suggesting that declines in whale shark populations are still occurring [16,19–22]. Such declines might be a result of illegal fisheries [23,24], other anthropogenic sources of mortality such as boat-strike and pollution [25], or by-catch as part of tuna purse-seine operations [26].

Despite being largely oceanic, whale sharks form ephemeral aggregations in coastal waters for a limited period of their life cycle where they are frequently seen at the surface [13]. The accessibility of such aggregations for researchers offers an opportunity to collect demographic data based on the identity, number and size of individuals. However, the outcomes of modelling studies of population trends based on metrics such as body size (mean total length) have been contradictory, even at the same aggregation site [20,27]. A key issue underlying this problem has been that trends observed in mean lengths can be due to changes in the abundance of size classes at either end of the distribution (i.e. both the gain or loss of the largest individuals and changes in recruitment to the aggregation that result in an increase or decrease in the numbers of smaller sharks). Disentangling these potential drivers of patterns in mean body length is therefore challenging [20]. However, using documented body sizes at maturity [28], quantification of the number of larger individuals in each subpopulation can be used to infer the portion of individuals that are mature or close to maturity. In turn, such assessment is a central part of the criteria for the designation of the conservation status of a species by the IUCN (criterion C—number of mature individuals; www.iucnredlist.org).

Whale sharks are generally thought to attain maturity at total lengths greater than or equal to 8 m, with 95% of the sharks at Ningaloo Reef [28], and 50% of the sharks off the east coast of Southern Africa [29] being mature at approximately 9 m length. Because logistic and ethical considerations prevent capture and restraint of these large animals, total lengths are typically estimated while sharks are free-swimming. A common technique is to estimate the size of the whale shark by comparing it visually with an object of known size, such as a boat, or a person swimming in close proximity to the animal [28,30,31]. Given that human spatial perception is biased underwater and encounters can be fleeting, such length estimates are likely to include considerable error even when made by experienced observers [32]. Consequently, before being able to draw conclusions on population trajectories based on metrics such as size, we must first identify and quantify observer errors and biases in size estimates. Here, we compare size estimates derived using visual and stereo-video techniques on the same whale sharks at Ningaloo Reef, Western Australia. The stereo-video technique provides a more accurate estimate of fish size [33,34] than visual techniques and thus allows the error and bias in visual estimates to be quantified. In turn, based on known lengths at maturity, this enables us to obtain a better estimate of the proportion of the population that is mature or close to maturity at this locality. We then compile all total length data publicly available for whale sharks from aggregations spanning their entire geographical range (Indian, Pacific and Atlantic Ocean basins) to provide an overview of the ranges of total length observed in recent decades (1995–2013).

## 2. Methods

We collected length data of whale sharks in nearshore waters at Point Cloates, Ningaloo Reef, Western Australia (22.67°S, 113.65°E), in four separate field trips (approx. 10 days each) between 2009 and 2011 during the peak period of the whale shark aggregation [35]. We used a spotter plane from 10.00 to 16.00 to locate whale sharks swimming at, or near the surface, off the reef front. The pilot directed our research vessel to the immediate vicinity of the sharks where snorkellers entered the water. While swimming with the shark, we (i) took identification photos of the flank above each pectoral fin from the fifth gill slit to the posterior point of the pectoral fin [36], (ii) determined the sex and assessed maturity status of male sharks by observing the presence/absence of claspers on the pelvic fins (whenever the shark stayed near the surface long enough for close inspection) and if present, by examining their length and thickness (if thick claspers extended past the pelvic fins, the shark was classified as mature), and (iii) estimated total length both by comparing the size of the shark with a biopsy spear (2.00 m) carried at all times by the same snorkeller (a strong swimmer and experienced free-diver), and by filming using a stereo-video camera system (www.seagis.com.au). Visual estimates of the total length of the shark were independently determined by each of three experienced researchers using the 2.00 m spear as comparison. After reconvening at the boat, all researchers discussed the visual estimate for the shark's total length, and a final value was agreed with an approximate precision of 0.5 m. The stereo-video camera system consisted of a pair of video cameras in underwater housings mounted on a bar configured to optimize the area of overlap of the field of view of the cameras. A diode unit was mounted in front of the cameras to allow synchronization of the frames of the video footage during analysis. We calibrated the camera system prior to each field trip following standard procedures [37–39]; however, we tested validation for a larger range of lengths than usually considered (i.e. we used test lengths of: 1, 3 and 5 m to validate the calibration and provide an estimate of measurement accuracy; electronic supplementary material, table S1) in order to account for the expected larger sizes of whale sharks (3–10 m) [40]. Such underwater stereo-video systems are known to generate absolute errors of around 1% of the measured length when using well-defined targets [34]. After collecting our whale shark stereo-video footage in the field, we used all the measurements that resulted in a precision of 5% of the measurement length (precision estimate provided by the software). We analysed the digital paired-images collected during the field trips with PhotoMeasure software (www.seagis.com.au). This software accounts for variation in angle and distance to the shark without the need for a scale reference in images, and allows direct calculation of total length from images capturing the entire shark in the field of view, avoiding the need to extrapolate. We considered the total length of the shark as the straight line measured from snout to the end of the caudal fin. The end of the caudal fin was estimated by drawing a line between the tip of the upper and lower lobes when the caudal fin was at the midpoint between strokes. Matching of identification photos using I3S software (Interactive individual identification; http://www.reijns.com/i3s) [41] showed that the same individual was sometimes encountered on more than one occasion. In the few cases when this occurred, we averaged stereo-video measurements (differing less than 5% from each other) from different sightings of the same shark in the same year prior to analysis, while keeping all the repeated visual estimates.

We applied linear mixed-effects models to our paired dataset of visual and stereo-video measurements (data in electronic supplementary material) to determine if the accuracy of visual estimates of total length varied with the shark size (as measured by the stereo-video). We used the absolute difference (*diff*) between lengths obtained visually (*visual*) and measured using the stereo-video as a response variable. After checking Spearman's collinearity, we included several combinations of the following non-collinear predictors in the model set: (i) *day*; (ii) time of the sighting to account for observer fatigue (*time*); (iii) the visual estimate of length of the shark sighted immediately prior to the encounter (*previsual*) to account for any bias associated with comparisons between sizes of sharks seen in successive encounters; and (iv) the total length obtained by stereo-video measurement (*TLN*). To account for variation in visual estimates of length for each individual shark, we included shark identity (*ID*) as a random effect. The sex of the shark was not included as a predictor, because we were interested only in the comparison between lengths estimated visually and by stereo-video. There were also many individuals (approx. 20%) for which sex could not be determined. We developed models using the *lmer* function from the lme4 package [42] in R [43]. We compared each model in the set using the weights (*w*AIC_c_ and *w*BIC) of two bias-corrected indices of parsimony: the Akaike's information criterion corrected for small sample sizes (AICc) and the Bayesian information criterion (BIC) [44]. To quantify the goodness of fit of each model, we used the marginal ($R_{m}^{2}$) and conditional ($R_{c}^{2}$) *R*^2^~[45].

We also compared the mean total lengths estimated by each method (visual and stereo-video). To determine the possible range of differences between mean lengths estimated by both methods, we used a resampling procedure with 10 000 iterations from each dataset and calculated the difference at each iteration [46]. We also used a bootstrapping procedure to estimate the mean standard error in our sample of stereo-video measurements. We resampled the stereo-video measurements 10 000 times with replacement, recalculating the bootstrap mean after each iteration and the standard error of the iterated means. This provided an assessment of the average difference between our mean estimates of length obtained by stereo-measurement from the mean length of the entire subpopulation of whale sharks visiting Ningaloo Reef.

We then compiled available data on total length of whale sharks from peer-reviewed publications. For the majority, total lengths were estimated using data collected in more than 1 year. When this was the case, we aggregated length data from each location and time period, and considered the length estimate to correspond to the last year of the period covered in the study. We report maximum, mean and minimum lengths available in the literature.

## 3. Results

**Figure 1.**
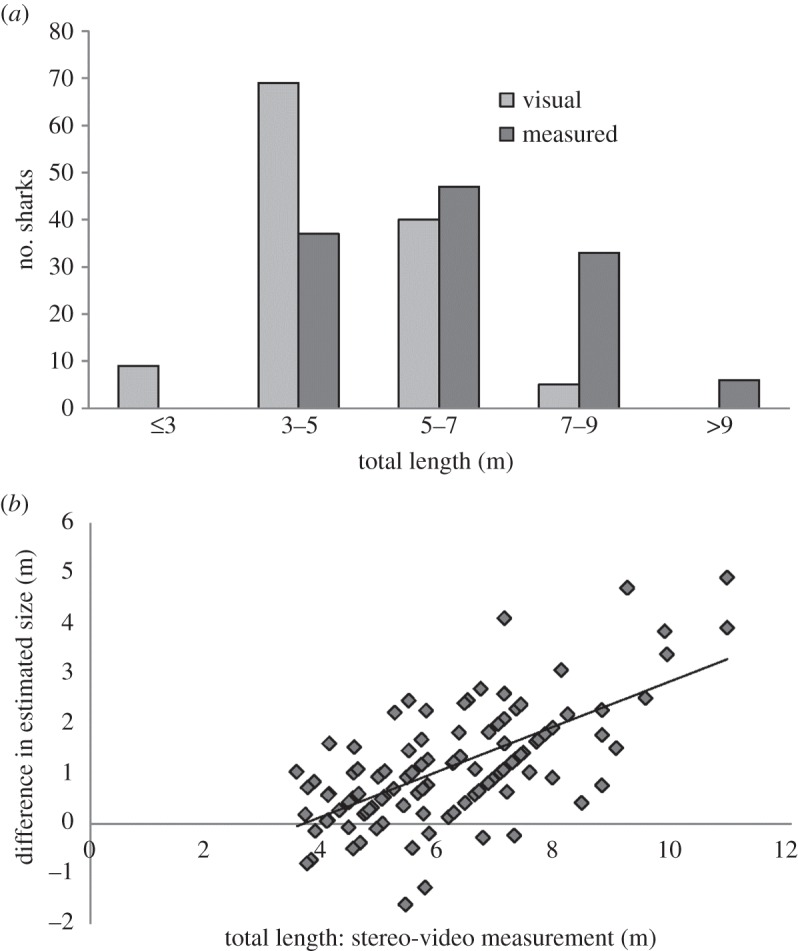
Whale shark length estimates. Assessment of differences in 123 length estimates of whale sharks sighted at Ningaloo Reef, Western Australia, during 2009–2011 obtained visually and by using more accurate methods (stereo-video camera system). (*a*) Number of whale shark sightings within each size category for each measurement technique: ‘visual’—visual assessment and ‘measured’—obtained using footage from the stereo-video camera system. (*b*) Differences between total lengths of whale sharks estimated visually and with the stereo-video camera as a function of the total lengths of whale sharks estimated with the stereo-video camera. Equation for regression line shown on the chart is *y* *=* 0.4509*x − *1.6429 (*R*^2^ = 0.4173).

We recorded a total of 311 sightings of whale sharks at Ningaloo Reef with 215, 50, 37 and nine sharks sighted in each field trip, respectively (including repeated sightings of some individuals). We obtained visual and stereo-video estimates of total length for 123 whale shark sightings corresponding to 95 individuals (i.e. excluding repeat sightings of the same individual within the same year) with a ratio of male: female: undetermined sex (M : F : U) of 65 : 11 : 19. Visual estimates of total length of whale sharks ranged between 2.5 and 8 m, with nine sightings having estimated total lengths less than or equal to 3 m, 69 sightings with lengths from 3 to 5 m, 40 sightings with lengths from 5 to 7 m and five sightings of sharks with lengths from 7 to 9 m (figure 1*a*); however, no visual estimate was greater than 8 m. The measurements we obtained from the stereo-video footage for the sightings of the same sharks (after the calibration procedure; electronic supplementary material, table S1) varied from 3.5 to 11 m, with 37 sightings of sharks from 3 to 5 m in total length, 47 sightings of sharks from 5 to 7 m, 33 sightings of sharks from 7 to 9 m and six sightings of sharks greater than or equal to 9 m in total length (figure 1*a*). All sharks were estimated to be larger than 3 m in total length when using the more accurate stereo-video method. Based on the morphology of claspers, only two of the 95 individuals were identified as mature males. One male had long claspers extending beyond the trailing edge of the pelvic fins, whereas the claspers of the other were ‘cauliflowered’ in appearance, possibly indicating previous sexual activity [28]. Visual estimates of the total length of these sharks were 6.5 and 8 m, whereas our stereo-measurements of the same sharks were close to 9 m (i.e. 8.76 and 8.41 m, respectively). These measured lengths were within the known range for size at maturity of whale sharks in the Ningaloo subpopulation [28].

The differences between lengths estimated visually and from stereo-measurements generally increased with shark size (figure 1*b*) with the largest discrepancies (between 3 and 5 m) for sharks more than 8 m in total length. Only on 13 occasions (approx. 10%) were the visual estimates larger than those made from stereo-measurements. In those cases, differences averaged 0.52 ± 0.47 m with the largest differences (1.27 and 1.61 m) recorded for two sharks measured by stereo-video to be approximately 6 m total length (figure 1*b*). When describing differences between lengths obtained visually and by stereo-video measurement, the model that obtained the highest *w*AIC*_c_* and *w*BIC support (95% and 98%, respectively; table 1) included total length obtained from stereo-measurements (*TLN*) and a random effect for individual (*ID*). Goodness of fit was also highest for this model ($R_{m}^{2}=43.5\%$ and $R_{c}^{2}=68.3\%$).

**Table 1.** Results of linear mixed-effects models. Ranked linear mixed-effects models of the absolute difference between the lengths estimated visually and by stereo-video measurements (*diff*) explained by the total length of sharks (*TLN*), *time* and *day*, and the random effect of individual (*ID*). Shown for each model are the bias-corrected weights of Akaike and Bayesian information criteria (*w*AIC_c_, corrected for small sample sizes and *w*BIC, respectively), and the marginal $\left(R_{m}^{2}\right)$ and conditional $\left(R_{c}^{2}\right)$ goodness of fit. Weights < 0.001 not shown.

model	*w*AIC*_c_*	*w*BIC	$R_{m}^{2}$	$R_{c}^{2}$
*diff* ∼ *TLN* + (1|*ID*)	0.986	0.999	43.2	68.3
*diff* ∼ *TLN* + *time* + (1|*ID*)	0.014	0.001	43.0	68.2
*diff* ∼ *TLN* + *time* + *day* + (1|*ID*)	—	—	43.0	69.2
*diff* ∼ *TLN* + *time* + *day* + *previsual* + (1|*ID*)	—	—	44.1	71.4
*diff* ∼ 1 + (1|*ID*)	—	—	0.0	65.4

**Figure 2.**
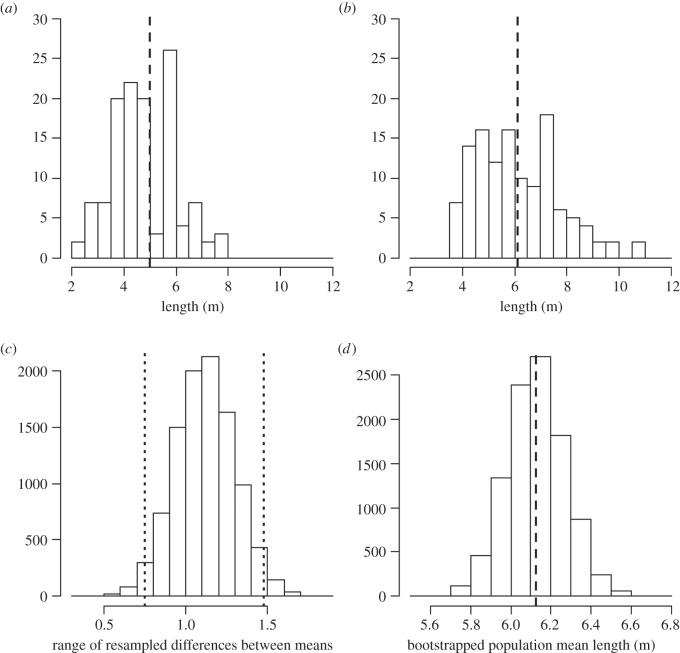
Frequency histograms of length estimates of whale sharks sighted at Ningaloo Reef, Western Australia (*n* = 123). Frequency histograms show: (*a*) total lengths estimated visually; (*b*) total lengths obtained via stereo-video measurement; (*c*) differences in the means between the two length estimation methods (visual and by stereo-video measurement) obtained with the resampling procedure (dashed-dotted lines indicate the range containing 95% of differences calculated and (*d*) bootstrapped means obtained using the stereo-video measurements data only. Dashed black lines in (*a*), (*b*) and (*d*) indicate mean total length. Vertical axes represent number of whale sharks measured in plots (*a*) and (*b*), and number of iterations used in our bootstrapping procedure in plots (*c*) and (*d*).

Based on our visual estimates of length, the mean size of whale sharks visiting Ningaloo each year was 5.002 m (s.d. ± 1.194 m; figure 2*a*). This compared with 6.128 m (s.d. ± 1.609 m; figure 2*b*) when lengths were estimated from the stereo-video. Our bootstrapping procedure compared the differences between the mean total lengths estimated by both methods and showed that 95% of the values were between 0.75 and 1.49 m (figure 2*c*). The mean length of whale sharks visiting Ningaloo Reef ranged from 5.6 to 6.6 in the 10 000 iterations of the bootstrapping resampling technique and averaged 6.126 ± 0.145 m (figure 2*d*).

**Figure 3.**
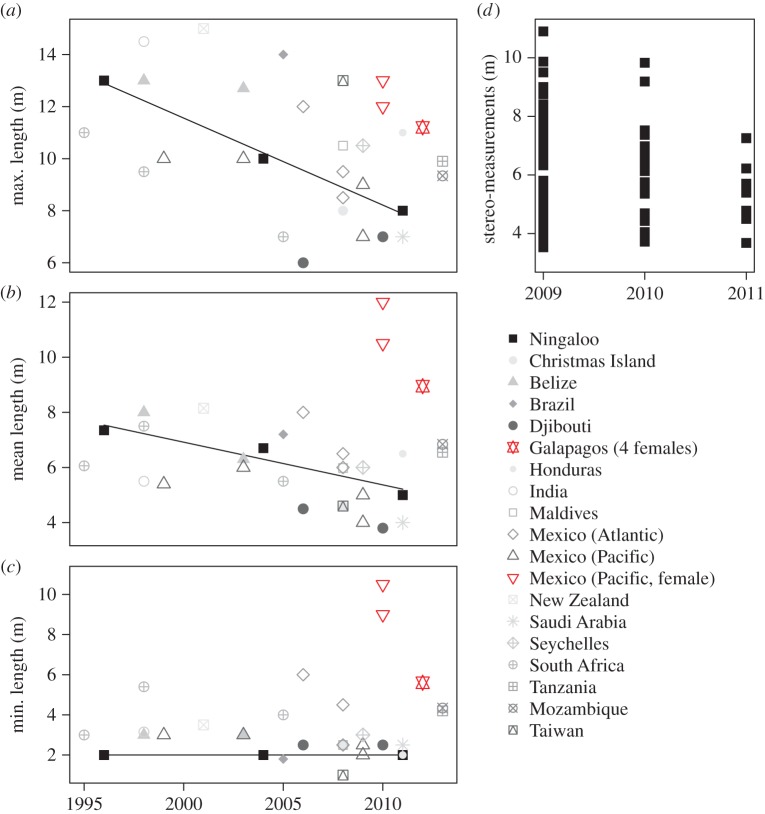
Reports of whale shark total lengths through time. Data are from 19 aggregations worldwide between 1995 and 2013, collected as detailed in table 2. Left panel: maximum (*a*), average (*b*) and minimum (*c*) total lengths published in the literature. Trend line refers to Ningaloo only where data from three different year groups were aggregated in three points and shown here only for the last year of sampling. Right panel: total lengths obtained from stereo-video measurements for each individual shark (points) we sighted at Ningaloo Reef between 2009 and 2011 (*d*). Note that in (*a*–*c*), measurements reported for Mozambique, Tanzania and Galapagos include values obtained using photogrammetry, which is known to be more precise than the visual estimates reported for most locations (notably prior to 2008 when most of the largest individuals were also recorded).

Figure 3 shows the range of lengths of whale sharks compiled from published studies worldwide, where data for maximum, mean and minimum length collected in each location were plotted for the last year of the period covered by the study (figure 3*a–c*). This figure shows that, except for large females observed at two locations in the East Pacific, the largest whale sharks (greater than 12 m total length) were only reported prior to 2008 and in only a few locations, including Ningaloo Reef. While minimum lengths reported by studies were highly variable (e.g. South Africa) and even relatively large for some locations (6 m in East Mexico; 4–5 m in Tanzania and Mozambique), estimated mean total lengths generally ranged between 5–8 m prior to 2006, and 4–7 m after 2006, again except in two locations in the East Pacific Ocean where large females have been reported [47–49]. This change in mean total length was evident despite most recent measurements in some locations (Mozambique, Tanzania, Seychelles, Djibouti, Mexico and Galapagos) using more accurate techniques (photogrammetry). For Ningaloo Reef, available visual estimates of length were averaged for three time periods: 1995–1996 [28], 2003–2004 [20,40] and 2009–2011 (this study; figure 1 and table 2). The largest sharks were observed in the first 2 years of the study at Ningaloo Reef, based on measurements obtained using the stereo-video method (figure 3*d*).

**Table 2.** Summary of available data for mean length and size range of whale sharks at aggregations worldwide. *N* is the number of individuals reported in each study and used to calculate mean total length in metres (m).

location	*n*	range (m)	mean (m)	period	references
Australia					
Christmas Island	82	2.5–8	4.6	2007–2008	[31]^d^
Ningaloo Reef	<500^a^	2–13	7.35^b^	1995–1996	[28]
	<500^a^	2–10	6.7	2003–2004	[20] and [40]
	123	2–8	5	2009–2011	this study
Belize					
Gladden Spit	25	3–13	8^c^	1998	[50]
	317	3–12.7	6.3	1998–2003	[30]
Brazil					
St Peter St Paul	54	1.8–14	7.2^a^	2000–2005	[51]
Djibouti					
Gulf of Tadjura	19	2.5–6	4.5	2006	[52]
	232	2.5–7	3.8	2003–2010	[53]
Ecuador					
Galapagos Islands	4	5.6–11.2^f^	8.95^e^	2011–2012	[48]
	82	4–13.1	11.35	2011–2013	[49]
Honduras					
Utila, Bay Islands	95	2–11	6.5	1999–2011	[54]
India					
Several locations	164	3.15–14.5	5.5	<1998	[55]^d^
Maldives					
South Ari Atoll	64	2.5–10.5	5.98	2006–2008	[56]
Mexico					
Gulf of California					
Bahía de Los Angeles	19	3–10	5.4	1999	[57]
	129	2.5–9	5	2003–2009	[47]
	30	3–10	6	<2003	[58]
Bahía de La Paz	125	2–7	4	2003–2009	[47]
Gorda Banks	15	9–12^f^	4	2003–2010	[47]
Island Espíritu Santo	8	10.5–13^f^	4	2003–2010	[47]
Gulf of Mexico	16	6–12	8	2006	[59]
Holbox	330	2.5–9.5	6^c^	2005–2008	[60]
Yucatan Peninsula	33	4.5–8.5	6.5^a^	2006–2008	[61]^e^
New Zealand					
North Island	26	3.5–15	8.15	1980–2001	[62]^d^
Saudi Arabia					
Al-Lith	64	2.5–7	4.00	2009–2011	[63]
Seychelles					
Mahe Island	549	3–10.5	6	2001–2009	[53]
Tanzania					
Mafia Island	56	4.20–9.90	6.55^e^	2012–2013	[29]^e^
Mozambique					
Praia do Tofu	123	4.34–9.34	6.84^e^	2010–2013	[29]^e^
South Africa					
KwaZulu-Natal	36	3–11	6.1^g^	1984–1995	[64]
	38	4–7	5.5^a^	2001–2005	[65]
	15	5.4–9.5	7.5^a^	1991–1998	[66]
Taiwan					
around Taiwan	597	1–13	4.6^c^	1995–2008	[67]

^a^Values estimated from size-frequency graphs presented in reference listed; in these cases, we calculated mean values using the upper limit of each size class in the original figure.

^b^Mean size estimated for 1997.

^c^Mean of range. Total lengths were estimated visually by comparison with an object of known length such as a snorkeller, boat or pole, unless otherwise indicated.

^d^Method not described in the reference.

^e^Included photogrammetry techniques to estimate the size of whale sharks.

^f^Also used visual comparison with vehicles on the beach (while doing aerial surveys).

^g^Size of whale sharks was obtained by measurement of vertebra growth rings.

## 4. Discussion

Errors in visual estimates were positively correlated with the size of the shark, so that as sharks increased in size, errors also increased. Additionally, there was a consistent bias in visual techniques towards the underestimation of the size of larger sharks (those individuals over 8 m total length). If a similar bias occurs at other aggregations, this has implications for our understanding of patterns of demography of whale sharks, because it suggests that the number of mature animals attending aggregation sites may be underestimated when using visual methods as a proxy for maturity assessment. At Ningaloo Reef, using visual techniques we did not identify any sharks with body lengths greater than 8 m, which corresponds to *L*_50_ size, i.e. the size at which 50% of the sharks at this locality were found to be mature [28]. In contrast using stereo-video measurements we identified more than 20 of the 95 individuals to be greater than 8 m in total length with 15 sharks less than 9 m (of which two were confirmed to be mature by visual examination of claspers) and six sharks greater than 9 m. Given our sample size of 95 individuals, which corresponds to 19–32% of the whale shark subpopulation visiting Ningaloo Reef (300–500 individuals) [40], and assuming that this is a representative sample of the subpopulation, our result suggested that about 6% (18–30) of the whale sharks visiting Ningaloo Reef were likely to be mature (greater than 9 m total length) and around 21% (63–105) were either mature or close to maturity (i.e. greater than 8 m total length).

Based on visual estimates, Bradshaw *et al.* [20] reported a reduction in the occurrence of larger sharks at Ningaloo Reef from the years 1995–1996 (size range: 2–13 m; approximately 29% of sighted sharks were greater than 8 m) to 2003–2004 (size range: 2–10 m; only approximately 5% were greater than 8 m). Our visual estimates of size for the period 2009–2011, which were comparable to those reported by Bradshaw *et al.* [20], recorded an even narrower range of lengths (only 2–8 m) with no sharks greater than 8 m in size. Given the issues of bias and error in visual techniques we identified, this trend in declining maximum size could be due to underestimates of the size of the largest animals increasing through time, although it is difficult to see why this should occur. Moreover, sampling effort from ecotourism operators has increased over time (owing to greater numbers of tourists), so that operators would be more likely to encounter larger animals at the upper edge of the size distribution in later years if they were present. Alternatively, if underestimates of size remained similar over time, the decline in the upper range of sizes might be a real trend, given that this bias would tend to obscure trends in maximum lengths over time. At present, it is not possible to distinguish between these hypotheses.

If the trend of declining maximum size of whale sharks at Ningaloo Reef is not an artefact of errors in visual measurements, this would be of potential concern for management and conservation because for many fishes, a shift in body lengths to smaller sizes is often associated with overexploitation and selective removal of larger individuals [3,4,11,68,69]. For whale sharks, any link to fisheries is not immediately obvious, because most targeted industries were prohibited over a decade ago [13]. However, a lagged effect of these practices or a greater extent of illegal fisheries for whale sharks than are currently recognized could account for this pattern. Lagged effects might also be expected given the *K*-selected life-history traits of the species, as any demographic consequences of fisheries could take several decades to be resolved. Additionally, other sources of mortality could be inadvertently promoting the loss of larger, mature individuals. These might include ship-strike or by-catch of whale sharks in purse-seine fisheries for tunas [26] which largely operate in the open ocean, where populations of adult sharks are thought to reside [13,30,67,70].

Because whale sharks are highly mobile, the shifts in body length of the species at Ningaloo Reef we observed could also be due to a change in the migratory pathways of the largest sharks associated with factors such as changes in oceanographic conditions [8,71,72]. However, this seems unlikely, given that we would expect to see both negative and positive changes in maximum sizes of sharks over time at different aggregations. Our meta-analysis of maximum total lengths of whale sharks from aggregations around the world revealed that sightings of the largest whale sharks were mostly reported prior to 2008 (except reports of females occurring in two locations in the East Pacific Ocean), a pattern consistent with our findings at Ningaloo Reef. This is further support for the idea that the declines in maximum size we recorded were not artefacts of the sampling technique.

Despite the error and bias associated with length estimates obtained using visual techniques, these still provided a reasonable approximation of mean total lengths of sharks sampled at Ningaloo Reef. Mean total lengths estimated visually and with the stereo-video system were relatively similar, with 95% of differences between 0.77 and 1.48 m (less than 10–16.5% of the size of an 8–9 m adult shark). Assuming that these results are representative of other aggregation sites, this suggests that visual estimates of mean size could be used as a metric for comparisons of the composition of whale shark subpopulations across time and/or locations with similar demographic structures. This is the case for all aggregations in the Indian Ocean and some in the Atlantic, but not in the Gulf of California or Galapagos where aggregations mostly consists of large females [47,48]. Our study confirms earlier work using visual techniques that suggested that whale sharks attending the aggregation at Ningaloo Reef were mostly juvenile or subadult animals [20,28]; however, the number of larger sharks may be greater than previously thought.

The stereo-video system provided more accurate estimates of the size and population structure of the whale sharks at Ningaloo Reef than the use of visual techniques. Owing to developments in camera technology, current stereo-video systems are smaller than the model used for this study and are also lightweight (2–3 kg), making them easier to use while swimming with whale sharks. They offer some advantages over still or single video cameras that use laser pointers to estimate size [32] because they are very accurate [33] and frames can be selected from the video where the full animal is in view and not flexed during a tail stroke. Such imagery allows the direct measurement of total lengths and therefore avoids the need for extrapolation as in other techniques. The use of accurate techniques to measure the size of whale sharks at aggregation sites where individual animals can be resighted over long time periods (up to approx. 20 years at Ningaloo Reef) offers the possibility not only of collecting accurate size measurements of whale sharks, but also quantifying growth rates of these animals in the wild [32]. Both these attributes (body length and growth estimates) are key variables to assess demographics, are important for the calculation of rates of population resilience and recovery, and thus are essential for conservation and management planning for the species.

## Supplementary Material

Supplementary information on the calibration of the stereo-video cameras Table S1: Measurements obtained with the stereo-video calibration Table S2: Whale shark size data we collected off Ningaloo Reef, Western Australia

## Data accessibility

All data are provided in electronic supplementary material.
