# Supplementary material for: Error and bias in size estimates of whale sharks: implications for understanding demography
Source: R Soc Open Sci. 2016 Mar 23;3(3):150668. doi: 10.1098/rsos.150668 (PMC4821267; doi:10.1098/rsos.150668)
Supplement: Supplementary information on the calibration of the stereo-video cameras Table S1: Measurements obtained with the stereo-video calibration Table S2: Whale shark size data we collected off Ningaloo Reef, Western Australia [file rsos150668supp1.doc]

# Supplementary Information

## Calibration of the stereo-video cameras

Toe calibrate the stereo-video camera, we distributed 50 targets in an approximate 2.2 m square (as shown in Figure below, left). This calibration range was adapted to suit both the size of the whale sharks and the average distance between the camera system and the whale sharks to be measured. During the calibration procedure, we determined the coordinates of the targets in the calibration range and their scale using a taper to measure several distances between targets at the extremities of the calibration range (see Figure on the right). We then captured stereo video footage of the calibration targets through a sequence of 20 different camera positions and orientations. The resulting imagery was processed using the CAL software package (www.seagis.com.au) in order to calibrate the stereo-video camera system.

| 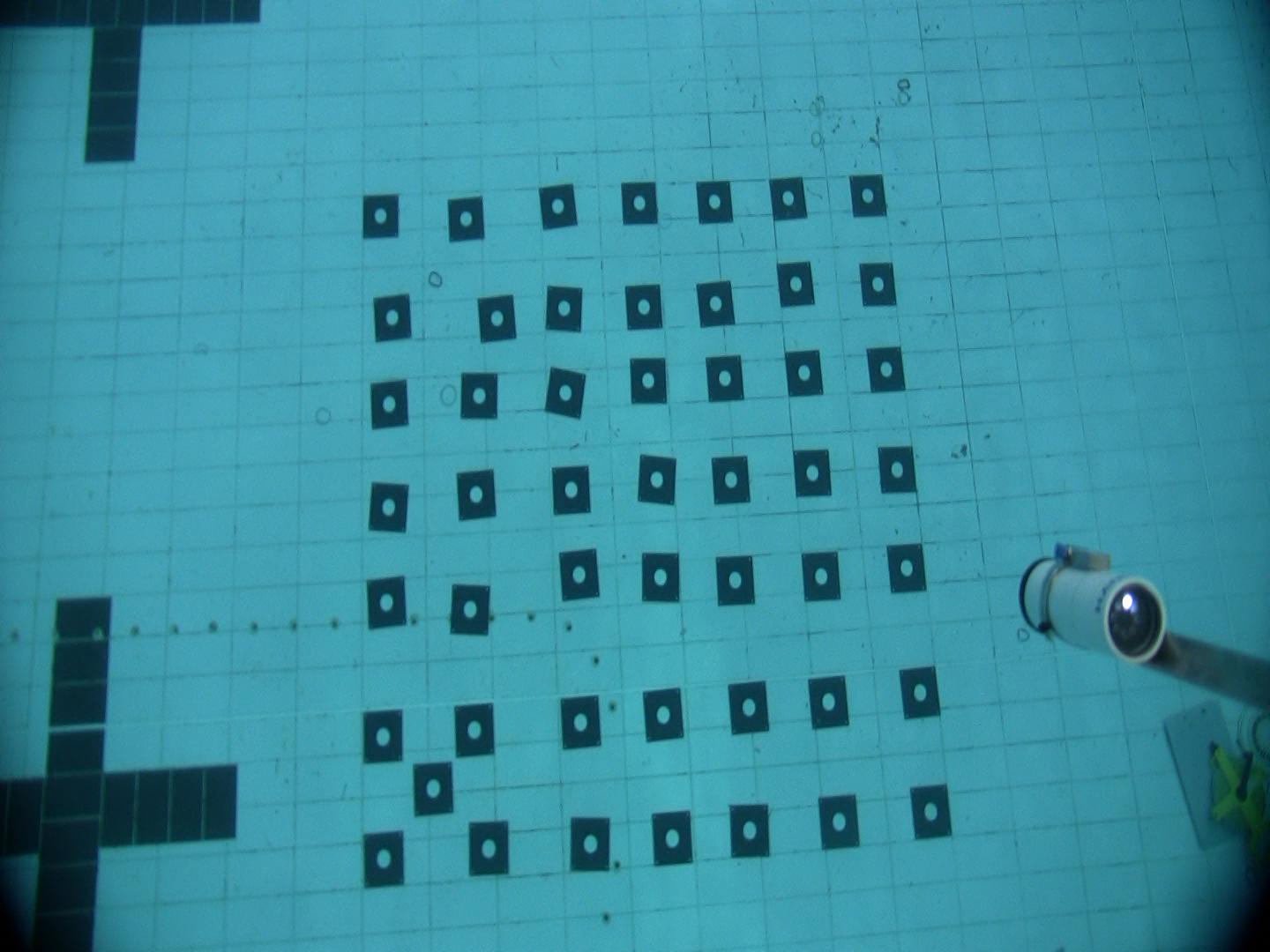 | 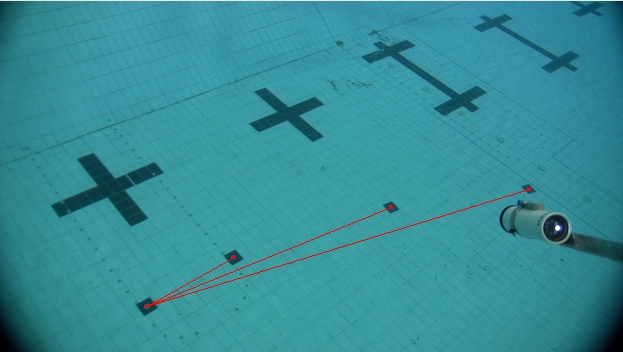 |
| --- | --- |

Images taken from the left stereo-video camera. The left image shows the array of calibration targets, the right image shows the test lengths measured after calibration for validation. Note the diode light in the lower right-hand side of the images to enable time synchronisation of the video footage

We validated and estimated accuracy of the calibration procedure by measuring a variety of test lengths (1, 3, and 5 m – maximum test length constrained by the maximum length we could include in view of sight of the cameras in the swimming pool) using the calibration targets and a tape measure (see image to the right). We collected stereo video footage of the test lengths and repeated measurements at 10 different camera positions and at various ranges between 3 and 8 m from the test lengths. Results are shown in Table S1.

## Table S1: Measurement obtained with the stereo-video calibration.

Measurements were made for three test lengths (1, 3 and 5 m) captured at 10 different camera positions at various ranges between 3 and 8 m from targets. Shown are the mean and standard deviation of each measurements, and the error of the measurement when compared to the know test length. Units are in meters.

| **Test length** | **Measurement** | **SD** | ***Error*** |
| --- | --- | --- | --- |
| 1 | 0.9949 | 0.002 | 0.005 |
| 3 | 2.9858 | 0.003 | 0.014 |
| 5 | 4.9997 | 0.025 | 0.0003 |

## Table S2: Whale shark size data we collected off Ningaloo Reef, Western Australia. *Previsual* indicates the length visual estimate of the shark sighted immediately prior to the encounter to account for any bias associated with comparisons between sizes of sharks seen in successive encounters (as described in the main document). SV: stereo-video camera. Greyed cells highlight repeated sightings for which we kept the visual estimate but averaged the stereo-video measurement (measurement obtained in each repeat is indicated in parenthesis).

| **ID** | **Year** | **Day** | **Time** | **Sex** | ***Previsual*** | **Visual estimate** | **SV measurement** |
| --- | --- | --- | --- | --- | --- | --- | --- |
| 1 | 2010 | 1 | 14.05 | M | 3 | 3 | 4.52 |
| 2 | 2009 | 6 | 11.25 | U | 3.5 | 5 | 6.33 |
| 3 | 2010 | 2 | 14.04 | U | 3 | 4 | 6.68 |
| 4 | 2011 | 1 | 14.00 | U | 6.5 | 5 | 6.22 |
| 5 | 2010 | 2 | 14.04 | M | 4 | 3 | 3.72 |
| 6 | 2011 | 2 | 13.32 | U | 3.5 | 3.5 | 3.68 |
| 7 | 2009 | 2 | 13.35 | M | 3 | 5 | 5.00 (5.03) |
| 7 | 2009 | 10 | 12.45 | M | 4.5 | 4.5 | 5.00 (4.98) |
| 8 | 2011 | 3 | 13.11 | M | 5.5 | 7.5 | 7.26 |
| 9 | 2009 | 2 | 13.45 | M | 5 | 7.5 | 9.00 |
| 10 | 2011 | 3 | 13.52 | M | 7.5 | 4.5 | 4.73 |
| 11 | 2009 | 2 | 13.50 | M | 7.5 | 6 | 6.88 |
| 12 | 2010 | 4 | 11.53 | U | 4 | 4.5 | 4.69 |
| 13 | 2011 | 3 | 14.31 | M | 4.5 | 4.5 | 5.51 |
| 14 | 2009 | 2 | 14.15 | M | 6 | 6 | 7.02 |
| 15 | 2010 | 4 | 11.59 | M | 4.5 | 4.5 | 6.31 |
| 16 | 2011 | 5 | 12.07 | M | 4 | 4.5 | 4.78 |
| 17 | 2011 | 8 | 11.09 | M | 6 | 7 | 5.39 |
| 18 | 2010 | 4 | 13.02 | U | 4 | 5 | 6.20 |
| 19 | 2011 | 9 | 12.07 | M | 9 | 5.5 | 5.70 (5.75) |
| 19 | 2011 | 9 | 13.07 | M | 5 | 5 | 5.70 (5.66) |
| 20 | 2009 | 3 | 12.46 | M | 6.5 | 5.5 | 6.58 |
| 21 | 2011 | 9 | 12.50 | U | 5 | 5 | 5.69 |
| 22 | 2009 | 3 | 13.04 | U | 6.5 | 3 | 5.21 |
| 23 | 2010 | 4 | 13.28 | M | 5 | 6 | 6.65 |
| 24 | 2009 | 3 | 13.10 | M | 3 | 3 | 5.45 (5.37) |
| 24 | 2009 | 3 | 13.20 | M | 3 | 3 | 5.45 (5.61) |
| 24 | 2009 | 8 | 11.45 | M | 6 | 4 | 5.45 (5.39) |
| 24 | 2009 | 8 | 11.45 | M | 8 | 4.5 | 5.45 (5.48) |
| 24 | 2009 | 9 | 13.25 | M | 6 | 4.5 | 5.45 (5.38) |
| 25 | 2010 | 4 | 13.40 | M | 6 | 4 | 5.67 |
| 26 | 2009 | 3 | 13.53 | M | 7 | 4 | 4.50 |
| 27 | 2009 | 3 | 14.14 | M | 4 | 7 | 9.50 |
| 28 | 2010 | 5 | 11.09 | M | 4 | 5 | 7.37 (7.47) |
| 28 | 2010 | 5 | 14.22 | M | 6 | 6 | 7.37 (7.27) |
| 29 | 2009 | 4 | 10.40 | M | 5 | 5 | 5.77 (5.72) |
| 29 | 2009 | 4 | 12.10 | M | 6 | 4.5 | 5.77 (5.82) |
| 30 | 2009 | 4 | 11.20 | M | 5 | 4 | 4.97 |
| 31 | 2010 | 5 | 13.35 | M | 6 | 4 | 6.46 |
| 32 | 2009 | 9 | 15.55 | M | 5.5 | 3 | 8.17 (8.83) |
| 32 | 2009 | 10 | 11.35 | M | 7 | 5.5 | 8.17 (7.50) |
| 33 | 2011 | 2 | 15.30 | M | 7 | 6 | 6.22 |
| 34 | 2009 | 4 | 11.45 | M | 4 | 6 | 6.58 |
| 35 | 2010 | 5 | 13.36 | F | 3 | 6.5 | 7.52 |
| 36 | 2011 | 4 | 13.30 | U | 6 | 5 | 4.50 |
| 37 | 2009 | 4 | 12.25 | M | 4.5 | 6.5 | 8.76 (8.68) |
| 37 | 2009 | 10 | 12.50 | M | 4.5 | 7 | 8.76 (8.94) |
| 37 | 2009 | 11 | 12.25 | M | 6 | 8 | 8.76 (8.64) |
| 38 | 2010 | 5 | 13.55 | U | 6.5 | 6 | 9.83 |
| 39 | 2009 | 9 | 12.05 | M | 4 | 6 | 7.29 (7.29) |
| 39 | 2009 | 9 | 12.10 | M | 6 | 6 | 7.29 (7.10) |
| 39 | 2009 | 9 | 12.15 | M | 6 | 5 | 7.29 (7.49) |
| 40 | 2009 | 5 | 12.30 | M | 6 | 4.5 | 4.82 |
| 41 | 2010 | 9 | 11.15 | M | 4.5 | 4 | 4.43 |
| 42 | 2010 | 8 | 10.46 | M | 4 | 6 | 6.22 |
| 43 | 2009 | 5 | 13.15 | F | 6 | 3 | 3.83 |
| 44 | 2009 | 5 | 13.25 | M | 3 | 4 | 4.93 (4.82) |
| 44 | 2009 | 10 | 11.10 | M | 6 | 4 | 4.93 (5.03) |
| 45 | 2010 | 8 | 13.18 | U | 6 | 6 | 6.13 |
| 46 | 2009 | 5 | 13.35 | M | 4 | 6 | 7.07 |
| 47 | 2010 | 8 | 13.53 | U | 8 | 3.5 | 5.75 |
| 48 | 2009 | 5 | 13.45 | M | 5 | 4 | 4.26 |
| 49 | 2010 | 8 | 14.03 | U | 3.5 | 4 | 6.40 |
| 50 | 2010 | 8 | 15.07 | M | 5 | 5 | 5.36 |
| 51 | 2009 | 6 | 11.00 | F | 8 | 4.5 | 3.78 |
| 52 | 2010 | 9 | 10.18 | M | 5 | 5 | 6.98 |
| 53 | 2009 | 6 | 11.10 | F | 4.5 | 3.5 | 4.51 |
| 54 | 2010 | 9 | 11.04 | M | 5 | 4.5 | 9.19 |
| 55 | 2009 | 6 | 11.30 | U | 5 | 7 | 5.73 |
| 56 | 2009 | 6 | 11.45 | U | 7 | 6 | 6.81 |
| 57 | 2009 | 6 | 13.00 | M | 5 | 2.5 | 3.53 |
| 58 | 2010 | 9 | 12.28 | F | 5.5 | 4 | 4.05 |
| 59 | 2009 | 4 | 11.25 | U | 4 | 4.5 | 5.19 |
| 60 | 2009 | 6 | 13.10 | M | 7 | 6 | 6.41 |
| 61 | 2009 | 5 | 12.00 | M | 6 | 6 | 7.63 |
| 62 | 2009 | 6 | 13.15 | M | 2.5 | 4.5 | 3.71 |
| 63 | 2009 | 7 | 12.30 | M | 6 | 6 | 5.80 |
| 64 | 2009 | 8 | 10.55 | F | 6.5 | 7 | 10.90 (12.45) |
| 64 | 2009 | 9 | 11.15 | F | 4.5 | 6 | 10.90 (9.36) |
| 65 | 2009 | 8 | 11.20 | M | 7 | 8 | 8.41 (8.81) |
| 65 | 2009 | 8 | 15.10 | M | 5 | 8 | 8.41 (8.02) |
| 66 | 2009 | 8 | 12.00 | F | 4.5 | 5 | 6.82 |
| 67 | 2009 | 8 | 12.20 | M | 5 | 5 | 8.06 (8.35) |
| 67 | 2009 | 8 | 12.45 | M | 5 | 5 | 8.06 (7.77) |
| 68 | 2009 | 6 | 13.30 | U | 4.5 | 7 | 6.72 |
| 69 | 2009 | 8 | 14.40 | M | 6 | 5 | 7.08 |
| 70 | 2009 | 10 | 13.20 | M | 6 | 3.5 | 4.09 |
| 71 | 2009 | 8 | 13.30 | M | 6.5 | 6 | 7.41 (7.07) |
| 71 | 2009 | 8 | 14.00 | M | 4 | 6 | 7.41 (7.74) |
| 72 | 2009 | 8 | 15.20 | M | 5 | 6.5 | 7.13 |
| 73 | 2009 | 9 | 11.25 | M | 6 | 4.5 | 5.67 |
| 74 | 2009 | 9 | 15.35 | M | 4.5 | 4 | 4.46 |
| 75 | 2009 | 9 | 11.50 | F | 6 | 4 | 4.42 (4.54) |
| 75 | 2009 | 10 | 12.10 | F | 6.5 | 4.5 | 4.42 (4.29) |
| 76 | 2009 | 9 | 12.35 | M | 5 | 4.5 | 5.51 (5.54) |
| 76 | 2009 | 11 | 15.10 | M | 6 | 6 | 5.51 (5.48) |
| 77 | 2009 | 9 | 13.05 | F | 5 | 6 | 7.36 |
| 78 | 2009 | 9 | 13.40 | M | 4.5 | 6 | 7.22 |
| 79 | 2009 | 9 | 15.00 | M | 4 | 4.5 | 5.03 |
| 80 | 2009 | 9 | 13.50 | M | 6 | 5 | 4.90 |
| 81 | 2009 | 10 | 11.00 | M | 3 | 6 | 8.17 |
| 82 | 2009 | 10 | 11.20 | M | 4 | 7 | 7.91 (7.72) |
| 82 | 2009 | 11 | 10.55 | M | 7 | 6 | 7.91 (8.11) |
| 83 | 2009 | 10 | 13.50 | M | 7 | 4 | 4.59 |
| 84 | 2009 | 10 | 13.15 | U | 7 | 6 | 7.67 |
| 85 | 2009 | 10 | 14.10 | M | 3.5 | 4.5 | 5.42 |
| 86 | 2009 | 11 | 11.30 | M | 5 | 4.5 | 4.99 |
| 87 | 2009 | 10 | 14.05 | F | 4 | 3.5 | 4.07 (3.98) |
| 87 | 2009 | 11 | 12.45 | F | 8 | 4 | 4.07 (4.02) |
| 87 | 2009 | 11 | 13.25 | F | 5 | 4 | 4.07 (4.21) |
| 88 | 2009 | 11 | 11.15 | M | 6 | 5 | 4.62 |
| 89 | 2009 | 11 | 11.45 | M | 4.5 | 4 | 3.85 |
| 90 | 2009 | 6 | 14.30 | U | 6 | 3.5 | 4.58 |
| 91 | 2009 | 8 | 10.15 | M | 3.5 | 6.5 | 9.87 |
| 92 | 2009 | 11 | 13.10 | M | 4 | 5 | 5.61 |
| 93 | 2009 | 10 | 12.20 | F | 4.5 | 4 | 5.04 |
| 94 | 2009 | 10 | 12.35 | M | 4 | 4.5 | 7.08 |
| 95 | 2009 | 9 | 11.40 | M | 4.5 | 6 | 7.78 |
